# A Case for Argument

**Published:** 1864-05

**Authors:** M. M. Eaton

**Affiliations:** Peoria, Ill.


### A CASE FOR ARGUMENT.

By M. M, EATON, Peoria, Ill.

October 30th, 1863, I was called to attend Mrs. R., a German lady, residing near this city. Having been previously consulted I knew that she was about to be confined, and I at once, in some haste, drove to her residence, being more prompt than usual on account of having been called some two years since to perform the operation of turning and delivering her —she having been under the care of Dr. ——, of this city, for about twenty hours before I was called. She had never had a living child though thrice pregnant.

As it happened, however, there was no need of haste, for I found the os uteri only slightly dilated. She was in a good condition every way so far as I could learn, and I left the case to nature. The pains increased, and after three hours, judge of my surprise, (after having examined her several times without detecting anything abnormal,) to feel what seemed to be a fœtus partly projecting through the os. I made slight traction and the mass slipped through, and truly it was a fœtus of about two months' development. The pains now ceased for an hour, when they came on and, in about three hours, she was delivered of a full-grown child, weighing eight pounds. The child gasped, but all efforts to establish respiration were fruitless. I took the fœtus home and placed it in alcohol, with my collections, it being to me a case of interest.

P. S.—Was she fecundated at two different times, or only once; the fœtus from some cause, being arrested in its development? The latter is my view. M. M. E.

---

The question of our correspondent was one of interest, the solution of which was surrounded with difficulties, while the Decidua was considered a new formation, completely closing the openings into the uterus. But by the demonstration that the decidua is the hypertrophied mucous lining of the uterus, the idea of the physical barrier to superfœtation has been removed. Numerous accidental causes may arrest or limit the nutrition of the fœtus in utero. And either of these circumstances will account for cases like that reported in the above communication, equally well.—[*Editors Journal.*]

